# Infrared laser therapy decreases systemic oxidative stress and inflammation in hypercholesterolemic mice with periodontitis

**DOI:** 10.1186/s12944-023-01934-9

**Published:** 2023-10-10

**Authors:** Isadora Martins Ribeiro, Maria Eduarda de Souza Barroso, Edgar Hell Kampke, Larissa Trarbach Figueiredo Braga, Bianca Prandi Campagnaro, Silvana dos Santos Meyrelles

**Affiliations:** 1 Graduate Program in Dental Sciences, Federal University of Espírito Santo (UFES), Vitória, 29.043-900 ES Brazil; 2 Graduate Program in Physiological Sciences, Federal University of Espírito Santo (UFES), Vitória, 29.043-900 ES Brazil; 3 Graduate Program in Pharmaceutical Sciences, Vila Velha University (UVV), Vila Velha, 29102-920 ES Brazil

**Keywords:** Photobiomodulation, Low-level light therapy, ApoE knockout, Periodontal disease, Hypercholesterolemia, Oxidative stress

## Abstract

**Background:**

Near-infrared irradiation photobiomodulation (NIR-PBM) has been successfully used in periodontal treatment as an adjuvant tool to locally improve cell function and regeneration. Although the relationship between periodontitis and systemic disease constitutes an important aspect of periodontal clinical research, the systemic effects of NIR-PBM in periodontitis are not well known. In this study, we aimed to investigate the effects of NIR-PBM on systemic oxidative stress and inflammation in an apolipoprotein E (ApoE) knockout mouse model of periodontal disease (PD).

**Methods:**

We evaluated alveolar bone loss by measuring the distance from the cementoenamel junction (CEJ) to the alveolar bone crest (ABC), reactive oxygen species (ROS) production in blood cells, inflammatory activity, plasma cholesterol levels, and lipid peroxidation levels in three experimental groups: (1) ApoEC, control group without intervention; (2) ApoEP, first molar ligation-induced periodontitis for 4 weeks; and (3) ApoEP + PBM, exposed to 808 nm continuous wave, ø ~ 3 mm2, 100 mW, 60 s of NIR-PBM for 7 consecutive days after 4 weeks of periodontitis. At the end of the experimental protocols, ApoEP mice presented significantly increased alveolar bone loss, ROS production, inflammatory activity, plasma cholesterol, and lipid peroxidation levels compared to the ApoEC group (*P* < 0.05). NIR-PBM for 7 days in the ApoEP + PBM mice significantly decreased systemic ROS production, inflammatory response, plasma cholesterol, and lipid peroxidation levels, similar to those found in the ApoEC group (*P* > 0.05). However, it was not capable of preventing alveolar bone loss (*P* > 0.05 compared to ApoEP mice).

**Conclusion:**

A 7-day treatment with NIR-PBM effectively reduces systemic oxidative stress and inflammatory parameters in hypercholesterolemic mice with PD. However, more studies with longer evaluation times are needed to confirm the systemic effects of locally applied NIR-PBM on PD associated with hypercholesterolemia.

## Introduction

Photobiomodulation (PBM) using near-infrared irradiation (NIR) is based on the theory that low-level light can modify and enhance cellular function [1]. The local reduction in edema and the decrease in oxidative stress markers and proinflammatory cytokines in PBM treatment are well established. However, some systemic effects, whereby light delivered to the body can positively impact distant tissues and organs, have also been reported [2]. Cellular effects attributed to NIR-PBM include an increase in adenosine triphosphate (ATP) production, a reduction in reactive oxygen species (ROS) production, protection against toxins, enhanced cell proliferation, and reduced apoptosis [3].

Excessive ROS production can lead to increased oxidative stress, resulting in tissue damage, lipid peroxidation, damage to deoxyribonucleic acid (DNA), protein damage, and the oxidation of important enzymes. However, ROS can also function as signaling molecules or mediators of inflammation [4].

Inflammatory mediators encompass a variety of soluble and diffusible molecules that act locally at the site of infection and at more distant locations. These mediators may be of endogenous origin (such as lipopolysaccharides from gram-negative bacteria) or exogenous (related to toxins and bacterial products) [5]. Increased oxidative stress plays a significant role in many human diseases, including periodontitis [6], hypercholesterolemia, atherosclerosis, chronic obstructive pulmonary disease, Alzheimer’s disease, and cancer [7].

Hypercholesterolemia is the most important modifiable risk factor for cardiovascular disease. Its reduction significantly decreases the risk of this type of disease in the population [8]. Moreover, high plasma cholesterol levels in hypercholesterolemic individuals reduce antioxidant activity and increase oxidative stress due to decreased superoxide dismutase (SOD) activity and increased malondialdehyde (MDA) activity, whereas the reverse is observed in normal individuals [9]. A study by Katz et al. [10] suggested that hypercholesterolemia could potentially serve as a link between chronic periodontal inflammation and atherosclerosis.

The relationship between periodontitis and systemic diseases constitutes an important area of clinical periodontal research. Periodontal disease (PD) may play a role in the development of a systemic inflammatory state by sharing inflammatory risk factors. However, systemic changes also affect oral health [11].

Periodontics has embraced laser technology in both surgical and nonsurgical treatments of periodontal tissues, either as a standalone treatment or as an adjunct, with many successful outcomes. Different lasers of high and low power have been utilized in periodontal treatments, offering benefits such as improved coagulation, antibacterial effects, root surface detoxification, removal of the smear layer, and enhanced bone recontouring [12].

While laser therapy has been employed in numerous studies related to periodontal disease (PD), its impact on systemic oxidative stress levels and inflammation in a hypercholesterolemia model of PD has not yet been explored. Therefore, our study aims to assess systemic levels of oxidative stress and inflammation in a hypercholesterolemic model, specifically using apolipoprotein E knockout (ApoE^−/−^) mice with PD subjected to the effects of NIR-PBM.

## Methodology

### Animals and experimental groups

**Fig. 1:**
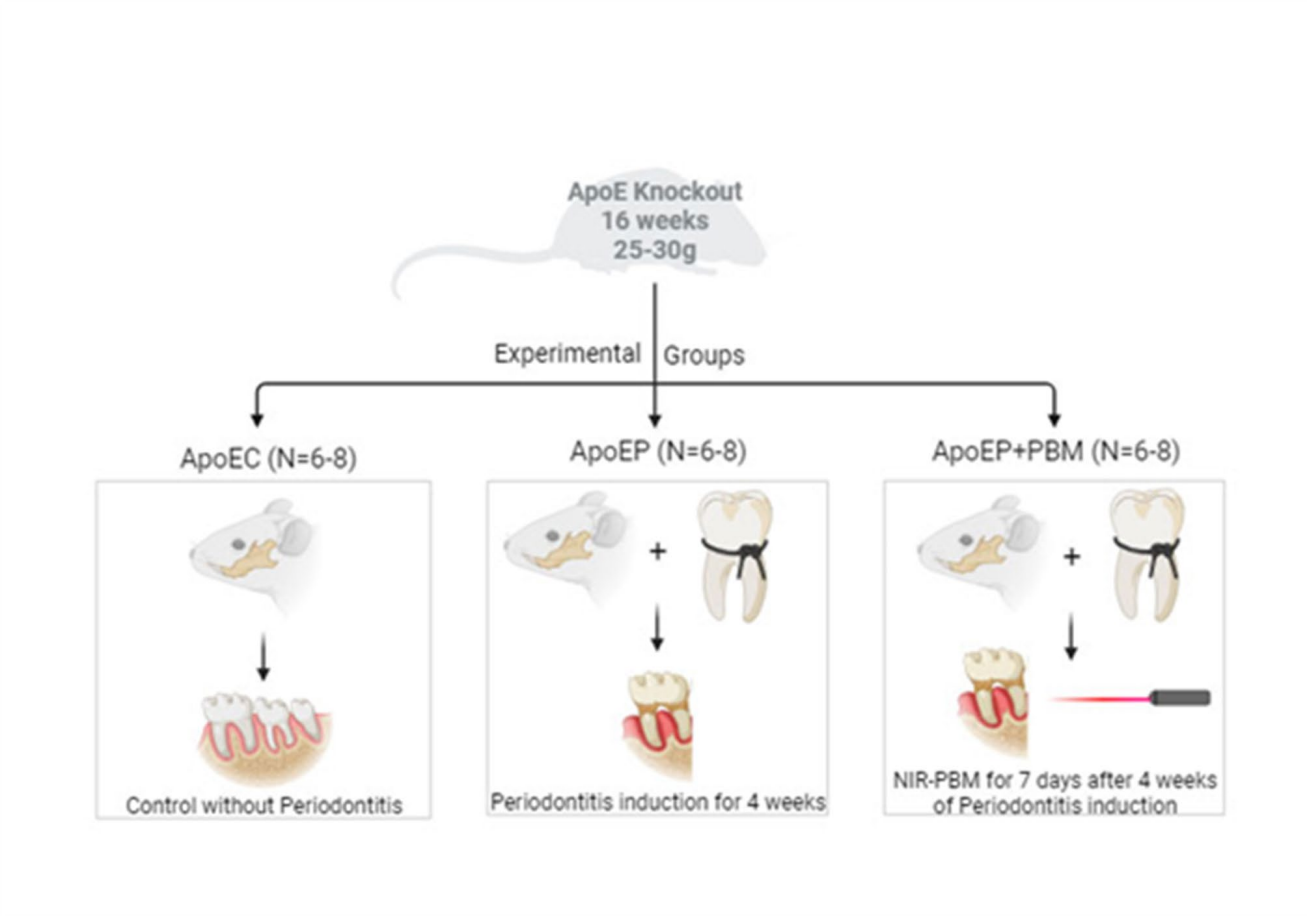
Schematic diagram of the experimental study design and the experimental groups. Apolipoprotein E knockout mice (ApoE^−/−^), including ApoEC (Control without Periodontitis induction), ApoEP (Periodontitis Induced for 4 weeks), and ApoEP + PBM (Periodontitis + 7 days of photobiomodulation)

The handling and care of the mice were conducted in accordance with the ethical principles outlined in the national and institutional guidelines for the care and use of laboratory animals, with approval from the ethics committee of Vila Velha University (No. 586–2021). ApoE^−/−^ mice, aged 16 weeks and weighing 25–30 g, were provided with a standard chow diet and had access to water ad libitum. They were individually housed in plastic cages under controlled conditions of temperature (22–23 °C), humidity (60%), and a 12-hour light/dark cycle. The mice were divided into three experimental groups: ApoEC (n = 6–8), which received no intervention; ApoEP (n = 6–8), where periodontitis was induced for 4 weeks; and ApoEP + PBM (n = 6–8), which underwent PBM treatment for 7 consecutive days after 4 weeks of periodontitis induction (Fig. 1).

### ApoE knockout mouse model

The ApoE knockout mouse model was developed by two laboratories in 1992 with the aim of creating better animal models for studying lipoprotein disorders and atherosclerosis and identifying genes that may modify atherogenesis and lesion progression [13, 14]. The increase in plasma cholesterol in ApoE knockout animals occurs due to the inhibition of the expression of the gene that encodes apolipoprotein E. Apolipoprotein E is a glycoprotein with a molecular weight of approximately 34 kDa that functions by binding to LDL (low-density lipoprotein) receptors to remove cholesterol from circulation in the liver. It is present in VLDL (Very-low-density lipoprotein), IDL (Intermediate low-density lipoprotein), and HDL (High-density lipoprotein). Its synthesis primarily occurs in the liver, but it is also produced in the brain and by macrophages [15].

To inhibit its expression, mouse embryonic stem cells are genetically modified by the insertion of two plasmids containing the neomycin resistance gene, which replaces part of the ApoE gene. These plasmids are inserted into blastomeres of wild-type mice (C57Bl/6), generating homozygous and heterozygous offspring. The crossing between homozygous animals results in ApoE knockout mice that present increased levels of VLDL in the plasma [13, 14].

Compared to other animal models of atherosclerosis, ApoE knockout mice have several advantages. They develop atherosclerosis spontaneously, without the need for a high-cholesterol diet [16, 17]. The development of atherosclerosis involves the activation of proinflammatory signaling, which includes the expression of cytokines and chemokines and promotes increased oxidative stress. Oxidative stress plays a crucial role in inflammatory responses, apoptosis, cell growth, changes in vascular tone, and LDL oxidation [18].

### Induction of periodontitis

Following the protocol for induction in mice suggested by Pereira et al. [19], with modifications, the mice were anesthetized with ketamine and xylazine (91 + 9.1 mg/kg) via intraperitoneal injection and positioned on an adapted surgical table that allowed for the opening of their oral cavity. Endodontic digital spacers #20 and #25 (Dentsply Maillefer, Ballaigues, Switzerland) with 3-mm bent tips were used to create space between the 1st and 2nd lower molars on the right side of the mice’s oral cavity. First, the #25 spacer was inserted, and after its removal, the #20 spacer was placed, with a 6.0 suture (Ethicon, USA) tied to its stem. Spacer #20 was then carefully removed to allow the suture thread to pass between the two teeth. Once the thread was inserted into the interproximal space, two knots were tied in the mesial area of the 1st molar to secure the ligature for plaque retention. The ligature was maintained in this region for a period of four weeks. After 4 weeks, the induction of periodontitis was confirmed by the presence of visualized plaque at the molar ligature suture and bleeding upon ligature suture removal and supragingival scraping to remove bacterial plaque [20].

### Laser irradiation

A Gallium Aluminum Arsenide diode laser (AsGaAl) known as Laser Duo (MMOptics Ltda, São Carlos, Brazil) was used for the PBM treatment. During the treatment, the mice were gently immobilized on their backs, and the laser was positioned in the extraoral region at the angle of the mandible, allowing the light beam to penetrate the entire intraoral region of the right lower molars of the animals. Laser treatment for the mice in the ApoEP + PBM group was conducted using an infrared wavelength and an energy density of 6 J per session (808 nm, continuous wave, ø ~ 3 mm², 100 mW) for a duration of 60 s. The treatment was administered for seven days with a 24-hour interval between sessions, totaling seven sessions. The ApoEP group received the same treatment, with the exception that the laser remained inactive. The ApoEC group received no intervention and served as the control. The mice were anesthetized 24 h after the conclusion of the laser treatment, and blood was collected through an intracardiac puncture. Subsequently, the mice were perfused with 10 ml of phosphate-buffered saline (PBS), and their mandibles were dissected [21].

### Mandible scanning electron microscopy and morphometric analysis

To confirm the establishment of PD and assess the effects of NIR-PBM, the mandibles of the mice were extracted and dissected. The organic tissue was removed from the samples by soaking them in 3% sodium hypochlorite for four weeks. After this period, the mandibles were rinsed in running distilled water for one minute. Subsequently, they were dried in an oven at 37 °C for seven days and stored in a humidity-free environment. To obtain scanning electron microscopy (SEM) images, the samples were left for 48 h at 50 °C and then coated with pure gold in a vacuum coater (Desk V, Denton Vacuum). The samples were then analyzed in direct mode using a scanning electron microscope (Jeol, JEM-6610 LV) [22].

After obtaining SEM images of the mandible, to assess alveolar bone loss, the linear distance in micrometers (μm) from the cementoenamel junction (CEJ) to the alveolar bone crest (ABC) of the mesial root, following the long axis of the tooth, was measured in the first mandibular molar using ImageJ, a public domain image analysis software. Bone loss was expressed in micrometres (μm) [23].

### Flow cytometry for ROS quantification

ROS production was assessed in blood cells by measuring intracellular superoxide anion (•O_2_^−^) and hydrogen peroxide (H_2_O_2_) using changes in median fluorescence intensity (MFI) emitted by dihydroethidine and dichlorofluorescein (DHE and DCF, Sigma‒Aldrich, USA, respectively). Briefly, 10^6^ cells were incubated with 160 mmol L^− 1^ DHE and 20 mmol L^− 1^ DCF at 37 °C for 30 min in the dark. The data were acquired using the FACSCanto II, and overlay histograms were analyzed using FACSDiva software by determining the average fluorescence intensity of 10,000 cells. Data were expressed as the median of emitted fluorescence intensity (MFI) [24].

### Biochemical analysis

Plasma cholesterol levels were assessed using a commercial colorimetric kit (Cholesterol Liquicolor - InVitro, Itabira/MG, Brazil). This kit utilizes reagents for the quantitative determination of cholesterol in plasma. The assay involves an enzymatic reagent – RGT (phosphate buffer, pH 6.5, 30 mmol/L; 4-aminoantipyrine, 0.3 mmol/L; phenol, 5 mmol/L; peroxidase > 5 KU/L; cholesterase > 150 U/L; cholesterol oxidase > 100 U/L; sodium azide, 0.05%) and a standard – STD (cholesterol, 200 mg/dL; sodium azide, 0.095%). Ten microliters (10 μL) of the plasma sample was mixed with 1000 μL of RGT, and 10 μL of STD was mixed with 1000 μL of RGT. They were then incubated for 5 min at 37 °C. Absorbances were measured using a spectrophotometer at a wavelength of 546 nm. To determine cholesterol values, the absorbance of the sample was divided by the absorbance of the STD and multiplied by 200. The plasma cholesterol values are expressed in mg/dL.

Inflammatory activity was assessed by measuring myeloperoxidase (MPO) activity. In this assay, hydrogen peroxide (H_2_O_2_) is cleaved by MPO, and the resulting oxygen radical reacts with O-dianisidine dihydrochloride, leading to the formation of a colored compound. Plasma samples (12 μL) were transferred to a flat-bottom microplate, and the biochemical reaction was initiated by adding 236 μL of O-dianisidine solution (comprising 16.7 mg O-dianisidine hydrochloride, 90 ml distilled water, 10 ml potassium phosphate buffer, and 50 μL H_2_O_2_ 1%). Absorbance was measured using an iMark® Absorbance ELISA microplate reader (Bio-Rad, Washington, USA) at a wavelength of 460 nm, with data recorded at 15-second intervals over a period of 10 min. The results were expressed as arbitrary units of myeloperoxidase activity (u.a. myeloperoxidase) as a function of time [25].

To determine oxidative stress, plasma lipid peroxidation was assessed using the TBARS assay. The generation of free radicals and lipid peroxidation are rapid processes, measured by their products, with thiobarbituric acid-reactive substances (TBARS), particularly malondialdehyde (MDA), being the primary indicator. To measure metabolites reactive to TBA, 43 μL of plasma was placed in a microtube with 7% perchloric acid and mixed using a vortex for 60 s. After this step, the samples were centrifuged at 7400 rpm for 10 min, resulting in the formation of a white pellet at the bottom of the microtube. Subsequently, 47 μL of the supernatant was transferred to two labeled microtubes, and an additional 53 μL of 0.6% thiobarbituric acid was added. The tubes were then placed in a thermocycler at 95 °C for 1 h. Following this incubation, the samples were centrifuged again and read in a spectrophotometer at 532 nm using a 96-well plate and the iMark® Absorbance Reader ELISA microplate reader (Bio-Rad, Washington, USA). The malondialdehyde (MDA) level was expressed in μM of MDA per milligram of protein [26].

### Statistical analyses

The results are expressed as the mean ± SEM. Normal distribution of the variables was assessed using the Shapiro‒Wilk test. When the results passed the normality test, the means of the values were statistically analyzed for comparisons among different groups using one-way ANOVA followed by Tukey’s post hoc test, conducted with GraphPad Prism Software, version 8.02 (GraphPad, Inc., San Diego, CA, USA). Differences were considered significant when *P* < 0.05 [27].

## Results

### Effects of PBM on periodontal disease

**Fig. 2:**
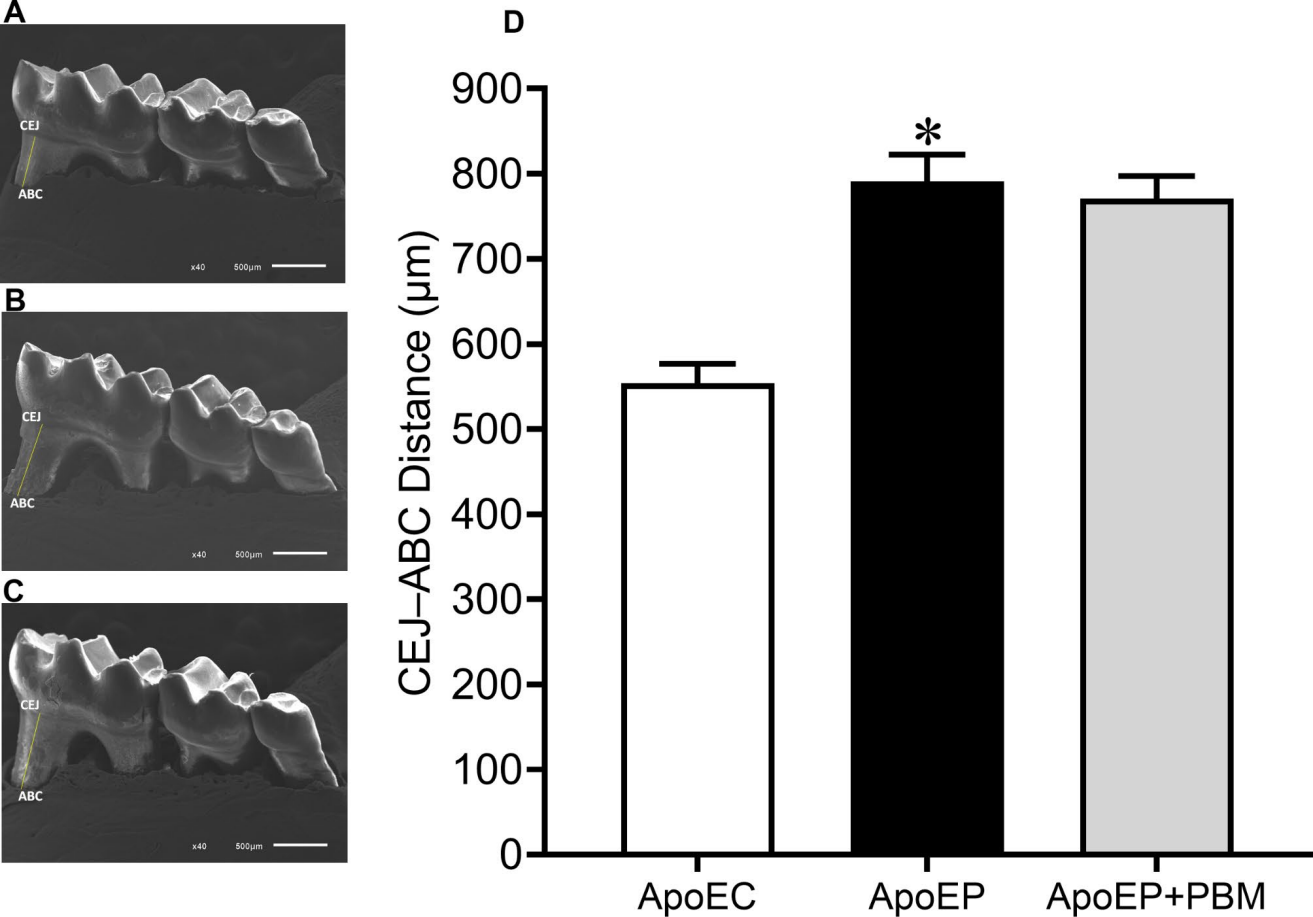
Typical scanning electron microscopy microphotographs showing the results of alveolar bone loss, measured in micrometers as the distance between the cementoenamel junction (CEJ) and the alveolar bone crest (ABC) in the experimental groups (40x objective, scale bar: 500 μm). (**A**) ApoEC (control, without periodontitis induction), (**B**) ApoEP (with 4 weeks of periodontitis), (**C**) ApoEP + PBM (with photobiomodulation for 7 consecutive days after 4 weeks of periodontitis), and (**D**) representative bar graph of alveolar bone loss in the experimental groups. Values are represented as the mean ± SEM (one-way ANOVA, Tukey’s post hoc test, n = 6–8 animals per group). *P < 0.05 vs. ApoEC mice

The success of ligature-induced periodontal disease (PD) was confirmed through significant alveolar bone loss in the ApoEP group compared to the ApoEC group (790.7 ± 31.90 μm vs. 553.8 ± 22.70 μm, *P* < 0.05, respectively). Interestingly, PBM treatment was unable to reverse bone loss in the ApoEP + PBM group (770.5 ± 27.03 μm, Fig. 2).

### Determination of plasma cholesterol

**Fig. 3:**
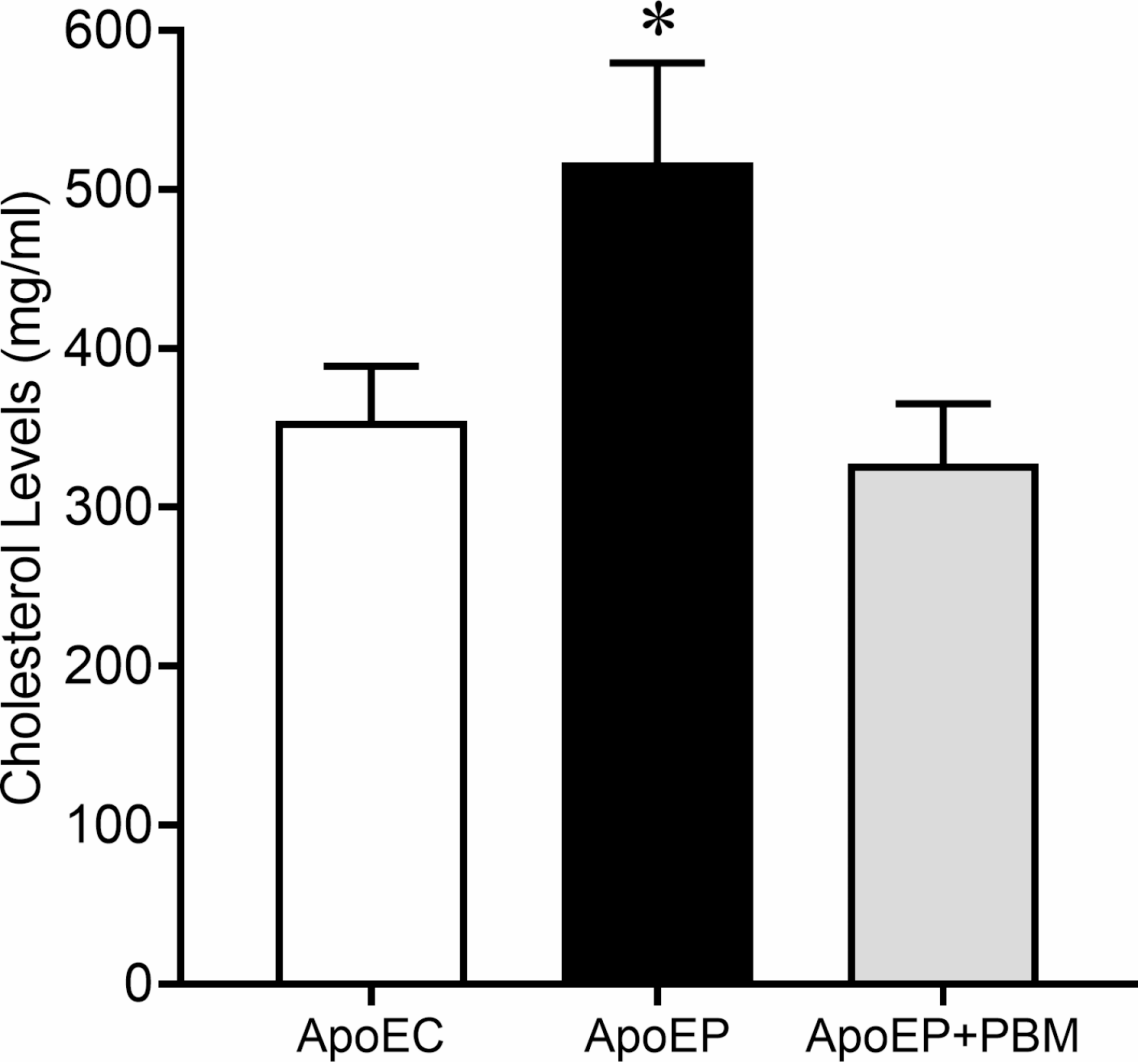
Plasma cholesterol measurement in the experimental groups. Values are represented as the mean ± SEM (one-way ANOVA, Tukey’s post hoc test, n = 6–8 animals per group). *P < 0.05 vs. ApoEC and ApoEP + PBM

Ligature-induced periodontitis significantly increased plasma cholesterol levels in the ApoEP group (516.9 ± 31.36 mg/ml, *P* < 0.05) compared with the ApoEC group (354.5 ± 34.26 mg/ml). NIR-PBM treatment decreased plasma cholesterol levels in the ApoEP + PBM group (327.6 ± 18.76 mg/ml) to ApoEC group values (Fig. 3).

### Analysis of myeloperoxidase (MPO) activity

**Fig. 4:**
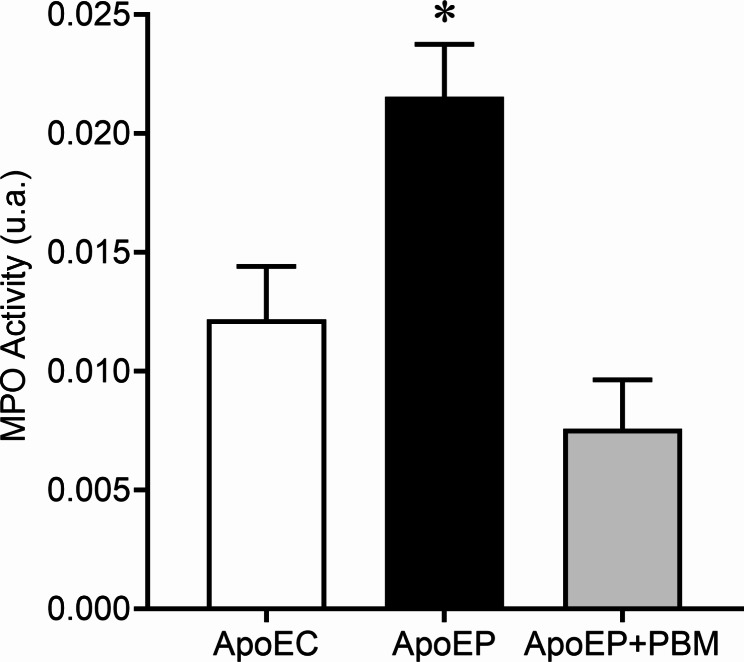
Proinflammatory enzyme myeloperoxidase (MPO) plasma activity. Values are represented as the mean ± SEM (one-way ANOVA, Tukey’s post hoc test, n = 6–8 animals per group). *P < 0.05 vs. ApoEC and ApoEP + PBM

The quantification of myeloperoxidase enzymes to assess inflammatory activity showed that the animals in the ApoEP group had higher levels of MPO (0.02153 ± 0.002214 a.u., *P* < 0.05.) when compared to those in the ApoEC group (0.01218 ± 0.002232 a.u.) and ApoEP + PBM (0.007571 ± 0.00263 a.u.). Moreover, mice in the ApoEP + PBM group had significantly decreased levels of MPO activity, similar to those found in the ApoEC mice, demonstrating a pronounced effect of PBM in controlling inflammatory activity in PD (Fig. 4).

### Superoxide and hydrogen peroxide levels

**Fig. 5:**
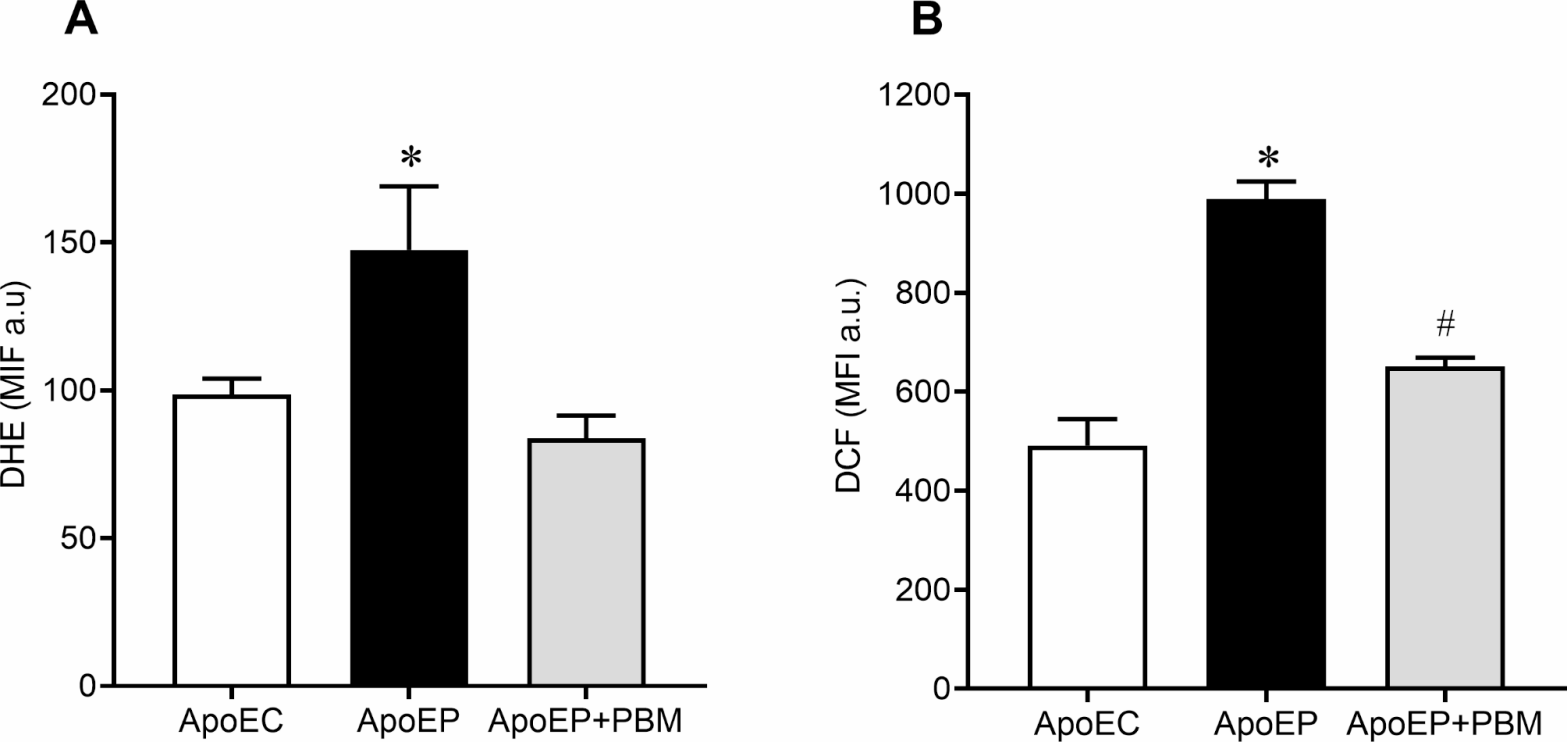
**A**) DHE and **B**) DCF levels in the experimental animals. Values are represented as the mean ± SEM (one-way ANOVA, Tukey’s post hoc test, n = 6–8 animals per group). *P < 0.05 vs. ApoEC and ApoEP + PBM; #*P* < 0.05 vs. ApoEC

The levels of superoxide anion and hydrogen peroxide, measured using the fluorescence markers DHE and DCF, respectively, showed that periodontitis significantly increased ROS production in ApoEP mice (147.5 ± 21.50 a.u.; 989.5 ± 35.50 a.u.; *P* < 0.05) compared to the ApoEC group (98.57 ± 5.331 a.u.; 491.7 ± 53.41 a.u.). NIR-PBM treatment restored DHE levels (83.75 ± 7.74 a.u.) to levels similar to those found in ApoEC mice. However, DCF levels in the ApoEP + PBM group decreased (651.3 ± 17.75 a.u.) compared to those in the ApoEP group but did not reach the levels observed in the ApoEC group (Fig. 5).

### Evaluation of lipid peroxidation levels

**Fig. 6:**
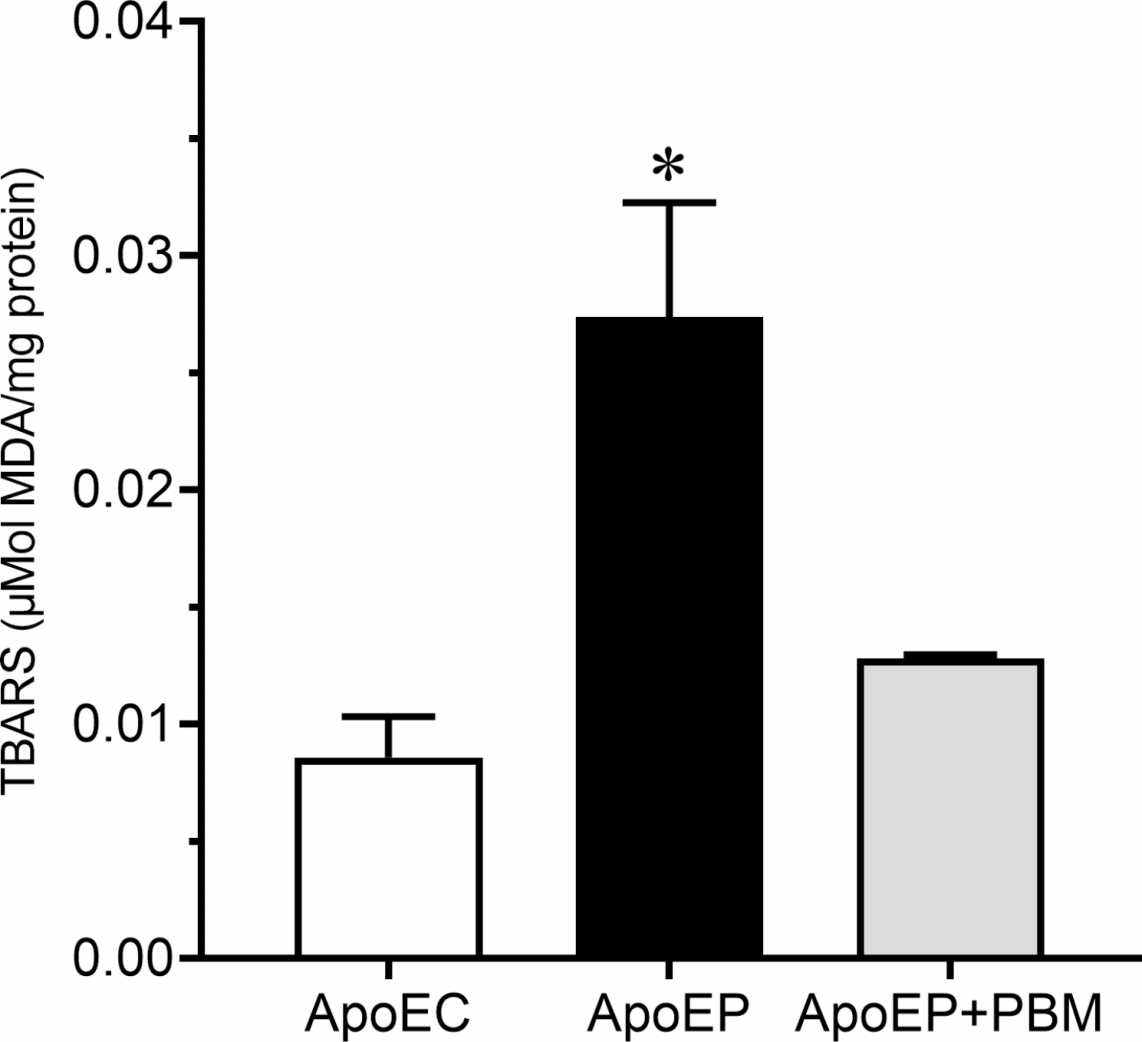
Evaluation of lipid peroxidation levels in ApoE hypercholesterolemic mice. Values are represented as the mean ± SEM (one-way ANOVA, Tukey’s post hoc test, n = 6–8 animals per group). *P < 0.05 vs. ApoEC and ApoEP + PBM

Animals from the ApoEP group showed higher lipid peroxidation levels (0.02738 ± 0.004885 μmol MDA/mg protein, *P* < 0.05) than those from the ApoEC group (0.008580 ± 0.001743 μmol MDA/mg protein). Moreover, the animals from the ApoEP + PBM group that received PBM treatment showed lower lipid peroxidation levels (0.01280 ± 0.0001581 μmol MDA/mg protein), similar to the untreated animals. These results indicate that periodontitis increases oxidative stress levels, but treatment with NIR-PBM is highly effective in reducing it (Fig. 6).

## Discussion

Our study evaluates the effect of photobiomodulation (PBM) on experimental ligation-induced periodontitis in hypercholesterolemic mice (ApoE knockout). Various types of lasers are available for periodontal treatment. PBM is a complex treatment, offering a wide range of combinations due to its extensive variation in parameters that can be utilized: wavelength, source power, energy density, power density, irradiation time, and total applied energy [28]. These variations have led to an increase in the number of published negative trials and have generated controversy, despite the large number of positive clinical results [29]. The photobiomodulatory effects of laser irradiation vary among different cell types, and this aspect has often been overlooked as a potential explanation for the conflicting results reported in the literature following treatment [30].

The PBM parameters used in our study were similar to those presented by Santos et al. [31], who employed a laser with a wavelength of 808 nm in a rat model of critical bone defect. Theodoro et al. [32] utilized the GaAlAs diode laser as monotherapy and as an adjuvant to mechanical treatment, using the same wavelength and power as in our study. However, in their work, they administered the treatment in a shorter timeframe and within a single session in rats with periodontitis. They observed a notable influence on the healing processes, tissue repair, and greater efficacy in modulating the inflammatory response in animals treated with laser, both in monotherapy and as an adjuvant treatment.

Several studies have shown that the application of diode lasers has bactericidal and detoxifying effects, and this technique demonstrates clinical benefits as an adjuvant to nonsurgical periodontal therapy [33, 34]. Low-intensity laser therapy promotes healing through collagen synthesis and angiogenesis [35].

In our study, after the removal of the ligature, supragingival scaling was performed to clean the tooth surfaces by removing plaque, and we administered 60 s of infrared laser treatment for seven consecutive days. It is worth noting that the literature often lacks comprehensive reports on all the parameters used, the number of sessions and the intervals between them [28]. The literature has shown that the combination of low-intensity laser therapy and root planing increases the effectiveness of periodontal disease treatment [20, 31, 36].

Our ligature-induced periodontitis model proved to be effective, according to our analyses of alveolar bone loss, which showed a decrease in bone level in animals that had induced periodontitis compared to those without periodontitis, as well established in the literature [19, 37].

Treatment with PBM, however, was ineffective in promoting bone neoformation in both the ApoEP and ApoEP + PBM groups in our study.

According to Tuner [28], PBM doses are cumulative, and several sessions in a short period can lead to inhibitory effects. In addition, Hablim et al. [2] stated that increasing PBM doses result in a cellular maximum response. If the dose exceeds the maximum value, the therapeutic effects of PBM will decrease and disappear, causing negative or inhibitory effects.

We chose the ApoE knockout mouse model, an animal model that presents high plasma cholesterol levels and develops atheromatous lesions very similar to those in humans, to evaluate the systemic effects of periodontitis and PBM. We observed higher levels of plasma cholesterol in ApoE mice with untreated periodontitis than in those that received PBM treatment. The literature reports that periodontitis can lead to a greater reservoir of cholesterol esters within macrophages and poses a significant risk for systemic implications, such as atherosclerosis [38, 39]. Moreover, bacterial products, cytokines, and chemokines resulting from the infectious and inflammatory periodontal process enter the bloodstream and may stimulate the upregulation of endothelial cell surface receptors, as well as adhesion expression on the vascular endothelium. This, in turn, leads to circulating monocytes adhering to the blood vessel endothelium. These monocytes migrate to the subendothelial space and differentiate into macrophages, which can take up oxidized low-density lipoprotein (LDL) and transform into foam cells, eventually leading to the apoptosis of LDL-laden macrophages. This process results in the accumulation of lipids in the subendothelial space, contributing to the formation of atheromatous plaques [40]. From this perspective, periodontitis may be considered a risk factor for cardiovascular diseases such as atherosclerosis [38]. Regarding the influence of the laser on cholesterol levels, the literature provides conflicting results, with some studies suggesting an increase [41], while others report a decrease [42].

Myeloperoxidase (MPO), a heme peroxidase found in large quantities in the azurophilic granules of neutrophils, serves multiple functions, including antimicrobial activity, participation in the biochemical pathway of ROS production, and serving as an important indicator for assessing neutrophil infiltration into tissues, oxidative stress, and tissue damage [4] and as a marker of inflammatory activity. Wei et al. [43] discovered that MPO levels in the periodontitis patient group significantly increased following periodontal clinical evaluation, emphasizing the pivotal role of ROS in periodontal tissue destruction. This finding aligns with the results of our study. Another study, conducted by Uslu et al. [44], evaluated the effect of diode laser treatment when applied as an adjunct to root scraping and planning in an experimental model of periodontitis. They concluded that diode laser treatment reduces inflammation and oxidative stress in periodontal tissues, a result similar to what we observed in our study, where we found a significant decrease in inflammatory activity in PBM-treated mice.

In our work, we assessed oxidative stress levels, which are positively associated with periodontitis [6]. During periodontitis, neutrophils release ROS in response to invading microorganisms. ROS are responsible for oxidative stress and contribute to much of the tissue damage during infection [45].

In our study, we quantified superoxide anion (O_2_^−^) and hydrogen peroxide (H_2_O_2_) levels by flow cytometry using DHE and DCF, respectively, as markers of ROS. We observed a significant increase in O2^−^ and H_2_O_2_ levels in the untreated periodontitis group. This increase was prevented in the NIR-PBM mice. However, previous research has indicated that low-intensity laser therapy can accelerate electron transfer (respiratory chain) and initiate ROS production, specifically increasing the production of superoxide anion, which can lead to cell damage [46].

Hablim et al. [2] raise the question of whether these types of ROS generated by PBM are identical to those naturally induced or not, with their benefits or harm depending on the rate at which they are produced. If superoxide is generated in the mitochondria at a rate that allows superoxide dismutase (SOD) to detoxify it into hydrogen peroxide, then H_2_O_2_ can diffuse out of the mitochondria to activate beneficial signaling pathways. However, if superoxide is generated at a rate or at levels beyond the capacity of SOD to handle, the accumulated superoxide can damage the mitochondria [2]. We also utilized the TBARS assay to evaluate the relationship between oxidative stress (lipid peroxidation), periodontitis, and PBM. Our study revealed a significant increase in lipid peroxidation levels in the ApoEP group when compared to the ApoEC group, while lipid peroxidation decreased in the APOE + PBM animals. The test detected malondialdehyde (MDA) formation resulting from the oxidation of lipid substrates [47]. During this process, ROS bind to polyunsaturated fatty acids, generating byproducts that can damage the membrane system, DNA, and cell proteins. Our results are consistent with a study by Almerich-Silla et al. [48], which observed significantly higher levels of MDA in periodontitis patients than in healthy controls. It has also been demonstrated that elevated serum and salivary MDA levels without changes in antioxidant status can lead to systemic and local complications in patients with periodontitis [49]. Increased lipid peroxidation has been found in the gingival fluid, plasma, and saliva of individuals with periodontitis [50, 51]. Others have also observed an association between PBM and reduced lipid peroxidation leading to a significant reduction in oxidative stress in cells and tissues, consistent with our findings [2, 52, 53].

### Study strengths and limitations

Our study is the first to demonstrate that NIR-PBM, as an adjunctive treatment for periodontal disease, has a significant beneficial effect in reducing systemic levels of cholesterol, inflammation, reactive oxygen species, and oxidative stress in a mouse model of periodontitis and hypercholesterolemia. These findings reinforce and support the importance of standardizing and including the use photobiomodulation therapy in dental clinical practice, especially, in situations where there is an associated chronic inflammatory systemic disease. However, some limitations of our study should be acknowledged. The wide range of NIR-PBM parameters in the existing literature can lead to contradictory results and could explain the absence of bone neoformation in the ApoEP + PBM group in our study. Additionally, more studies to evaluate the long-lasting systemic photobiomodulation effects need to be conducted, and finally, it’s worth noting that an ApoE knockout mouse model used in this study may not fully capture all the nuances of a hypercholesterolemic individual with periodontitis.

## Conclusion

In hypercholesterolemic mice with periodontitis, seven days of NIR-PBM treatment effectively reduced ROS production, plasma cholesterol, lipid peroxidation, and inflammatory activity. However, the observed benefits did not extend to bone formation, likely due to treatment duration and/or PBM dose. Our findings suggest that NIR-PBM has the potential to mitigate systemic factors in periodontal disease progression in hypercholesterolemic conditions. Future research with longer evaluation periods and varying doses is necessary to fully understand PBM’s impact on hypercholesterolemia-related periodontitis.

## Data Availability

The datasets utilized and/or analyzed during the current study are available from the corresponding author upon reasonable request.

## Abbreviations

µm: Micrometers
•O2−: Superoxide anion
ABC: Alveolar bone crest
ApoE-/-: Apolipoprotein E knockout
AsGaAl: Gallium Aluminum Arsenide
ATP: Adenosine triphosphate
CEJ: Cementoenamel junction
DCF: Dichlorofluorescein
DHE: Dihydroethidium
H2O2: Hydrogen peroxide
HDL: High-density lipoprotein
IDL: Intermediate low-density lipoprotein
J: Joules
LDL: Low density lipoprotein
MDA: Malondialdehyde
MFI: Median fluorescence intensity
MPO: Myeloperoxidase
NIR: Near infrared irradiation
PBS: Phosphate-buffered saline
PD: Periodontal disease
ROS: Reactive oxygen species
SEM: Scanning Electron Microscopy
SOD: Superoxide dismutase
TBA: Thiobarbituric acid
TBARS: Thiobarbituric acid-reactive substances
VLDL: Very-low-density lipoprotein
